# It is not enough to sing its praises: the very foundations of precision psychiatry may be scientifically unsound and require examination

**DOI:** 10.1017/S0033291721000167

**Published:** 2021-07

**Authors:** Jim van Os, Annemarie C. J. Kohne

**Affiliations:** 1 Department of Psychiatry, UMC Utrecht Brain Centre, University Medical Centre Utrecht, Utrecht University, Utrecht, The Netherlands; 2 Department of Psychiatry and Neuropsychology, School for Mental Health and Neuroscience, Maastricht University Medical Centre, Maastricht, The Netherlands; 3 Department of Psychosis Studies, Institute of Psychiatry, Psychology & Neuroscience, King's College London, London, UK; 4 Department of Psychiatry, Academic Medical Centre in Amsterdam, Amsterdam, The Netherlands

Reply to Salagre and Vieta

We thank Salagre and Vieta for their thoughtful and cogent comments. The admission that they are ‘in love’ with the concept of precision psychiatry, while in jest, does hit the nail on the head as it may be best summarized as exactly that: a collective overvalued belief that ‘biology plays a determinant role’ and that ‘mental disorders are disorders of the brain’ to be ‘tracked through biological clues’. In their reply, they reiterate the promise of precision psychiatry: how multi-omics, neuroimaging, big data and a range of high-density data approaches should converge towards specific biomarkers that can lead to biological stratification and, ultimately, to ‘precise’, person-specific treatments. They suggest that the types of fundamental concerns that we raise are best relegated to the realm of psychology. What is implied – without quite stating it – is that mental disorders (mental states) are expressions of brain pathology (physical states) and therefore should be studied under a linear model of body/brain-causes-mind. The issue of understanding possible ‘biomarkers’ of human emotions (as they frame our concern that it remains unknown if, how and to what degree mental phenomena are represented physically and – even if this were so – why this would be relevant to understanding mental suffering) is another concern altogether that they suggest takes us into ‘philosophical grounds’ which remain outside the scope of precision psychiatry.

## Is precision psychiatry self-evident?

We believe their reply is important as it forms part of what Braslow and colleagues refer to as ‘psychiatry's taken-for-granted, everyday beliefs’: that the *promise* of precise biology to remedy mental suffering is enough to make it self-evident (Braslow, Brekke, & Levenson, 2020). The implicit premise of precision psychiatry is that phenomena of the mind are physically represented and that these representations are relevant to our understanding of mental suffering. This belief is so strong that it does not require explicit reflection, let alone further examination. To belong to the traditional academic psychiatric community is to reiterate the self-evident nature of the belief. To seriously entertain the hypothesis that, for example, schizophrenia may not be a self-evident disorder of the brain is dismissed as ‘antipsychiatry’ (Sommer, Kahn, Denys, Schoevers, & Aleman, 2015).

However, not everybody agrees with such a stance. In fact, psychiatry is faced with increasingly blunt assessments of its reductionist belief system, written by eminent – mostly non-psychiatric – scholars in prestigious mainstream journals (some examples in Table 1). Since the civil rights movements of reformist psychiatry in the 1970s (later framed as ‘antipsychiatry’) and the ‘recovery movement’ in the 1980s, the patient's voice, and the focus on the existential domain of personal recovery, has not gained much traction in academic psychiatry, some even considering it a ‘hoax’ (Schizophrenia Research Forum, 28 November 2017). As a result, recovery-focussed work in the mental health care sector is hampered by limited ‘institutional readiness’ (Leamy et al., 2014) and by psychiatry's inability and/or unwillingness to escape the ‘epistemic prison’ of the ‘right medication for the right DSM-diagnosis’ (Gardner & Kleinman, 2019; Hyman, 2010). In reaction, novel and increasingly popular movements like *Mad in America* (MIA; www.madinamerica.com) produce a stream of articles focussed on debunking the same belief system that Salagre and Vieta passionately advocate. Although it is easy to dismiss MIA as unreasonable ‘antipsychiatry’ (Sommer et al., 2015), it may also be viewed as a campaign to address epistemic injustice (Crichton, Carel, & Kidd, 2017) brought about by the ‘myopia’ (Braslow et al., 2020) of an academic psychiatry that has unlearned to hear an individual's personal meaning in the experience of mental distress.

**Table 1.** Recent publications about how ‘precision psychiatry’ is viewed outside psychiatry

Citation	Authors
There is enormous investment in basic neuroscience research and intensive searches for informative biomarkers of treatment response and toxicity. The yield is close to nil. Much of the mental-health-related burden of disease may be induced or prevented by decisions in areas that have nothing to do with the brain. Our societies may need to consider more seriously the potential impact on mental health outcomes when making labour, education, financial and other social/political decisions at the workplace, state, country, and global levels.	Ioannidis (2019)
Ironically, although limitations of ‘biologic treatments’ are widely recognized, the prevailing message remains that the solution to psychological problems involves matching the ‘right’ diagnosis with the ‘right’ medication. Consequently, psychiatric diagnoses and medications proliferate under the banner of scientific medicine, although there is no comprehensive biologic understanding of either the causes or the treatments of psychiatric disorders.	Gardner and Kleinman (2019)
We suggest that clinical psychiatry's taken-for-granted, everyday beliefs, and practices about psychiatric disease and treatment have narrowed clinical vision, leaving clinicians unable to apprehend fundamental aspects of patients' experiences.	Braslow et al. (2020)
The main message delivered to lay people, however, is that mental disorders are brain diseases cured by scientifically designed medications. Here we describe how this misleading message is generated. Biomedical observations are often misrepresented in the scientific literature through various forms of data embellishment, publication biases favouring initial and positive studies, improper interpretations, and exaggerated conclusions. These misrepresentations are spread through mass media documents. Exacerbated competition, hyperspecialization, and the need to obtain funding for research projects might drive scientists to misrepresent their findings. These misrepresentations affect the care of patients.	Dumas-Mallet and Gonon (2020)

## AI solutionism

Apart from raising the model of *pathological-brain*/*body-causes-abnormal-mind* to the level of self-evidence, ignoring the ‘hard problem of consciousness', Salagre and Vieta assume another self-evident property of mental suffering: that it is determinable and predictable. Thus, they assume that there naturally will be such a thing as the ‘right treatment’ at the ‘right dose’ at the ‘right time’ for an individual with mental distress. In their view, analytical innovations such as machine learning will unravel determinability and predictability for use in clinical practice. However, this stance overlooks the underlying question to what degree mental suffering can be considered determinable and predictable in the first place – and therefore runs the risk of becoming a form of messaging called ‘AI solutionism’ (AI = artificial intelligence). This is the supposition that, as long as there is enough data, any human outcome can be computed based on machine learning algorithms (Chen & Asch, 2017). The question, however, is whether this can be considered a reasonable hypothesis in the case of mental outcomes.

AI solutionism holds that mental outcomes are determined – and therefore predictable. However, mental outcomes are unpredictable because they are inextricably linked to stochastic events in a complex system where chaos theory rules. Chaos theory describes the phenomenon that with the wisdom of hindsight all events may well be determined – but prospectively remain unpredictable. Although there are undoubtedly factors which have a statistical association with mental outcomes – for example, the loss of a loved one and the subsequent feeling of sorrow – the moment of losing a loved one at some point in time is unpredictable.

It is attractive to assume that, say, getting better on an antidepressant is a kind of determined process so that machine learning based on ‘everything’ can predict treatment response in a particular patient. However, the scientific basis for this is lacking, both conceptually and meta-analytically (e.g. Kennis et al., 2020). The reason for this was described by Tikhodeyev and Shcherbakova in the context of the mutagenic effect of ultraviolet radiation. Although the amount of mutagenic damage in microorganisms can be reliably predicted based on the amount of radiation, temperature, duration and culture medium, it cannot be predicted in which microorganism and where, in this microorganism, in the genome mutations will occur (Tikhodeyev & Shcherbakova, 2019). The explanation, according to chaos theory, is that even in the case of a deterministic (non-random) process, simple nonlinear systems cannot be predicted in the future. Machine learning cannot solve this (Chen & Asch, 2017).

The same applies to mental suffering: although there are weak therapeutic influences of factors at the group level, it remains unpredictable whether these influences lead to change in the stochastic ecosystem of a specific individual. Unpredictability in the ecosystem is partly due to the so-called butterfly effect: the sensitivity of the future to the most minute random change in the baseline condition. The importance of the butterfly effect is all the greater when one understands that ‘getting better’ on an antidepressant is largely dependent on a complex placebo-effect that has to do with expectation, relationship, being observed and time (Kirsch, 2014). Therefore, we fail to see why determinability and predictability should be considered self-evident postulates of precision psychiatry.

## Conclusion

In conclusion, the very foundations of the concept of precision psychiatry are unsafe. It is therefore not enough to merely sing its praises. Perhaps it would be more prudent to first focus on the scientific holes in the theory before building a practice that the world outside the culture of traditional academic psychiatry is increasingly unwilling to accept.
